# Transmembrane Transcription Regulators Are Widespread in Bacteria and Archaea

**DOI:** 10.1128/spectrum.00266-23

**Published:** 2023-05-08

**Authors:** Lucas M. Demey, Vadim M. Gumerov, Jiawei Xing, Igor B. Zhulin, Victor J. DiRita

**Affiliations:** a Department of Microbiology and Molecular Genetics, Michigan State University, East Lansing, Michigan, USA; b Department of Microbiology and Translational Data Analytics Institute, The Ohio State University, Columbus, Ohio, USA; South China Sea Institute of Oceanology

**Keywords:** cell membranes, signal transduction, transcription factors

## Abstract

To adapt and proliferate, bacteria must sense and respond to the ever-changing environment. Transmembrane transcription regulators (TTRs) are a family of one-component transcription regulators that respond to extracellular information and influence gene expression from the cytoplasmic membrane. How TTRs function to modulate expression of their target genes while localized to the cytoplasmic membrane remains poorly understood. In part, this is due to a lack of knowledge regarding the prevalence of TTRs among prokaryotes. Here, we show that TTRs are highly diverse and prevalent throughout bacteria and archaea. Our work demonstrates that TTRs are more common than previously appreciated and are enriched within specific bacterial and archaeal phyla and that many TTRs have unique transmembrane region properties that can facilitate association with detergent-resistant membranes.

**IMPORTANCE** One-component signal transduction systems are the major class of signal transduction systems among bacteria and are commonly cytoplasmic. TTRs are a group of unique one-component signal transduction systems that influence transcription from the cytoplasmic membrane. TTRs have been implicated in a wide array of biological pathways critical for both pathogens and human commensal organisms but were considered to be rare. Here, we demonstrate that TTRs are in fact highly diverse and broadly distributed in bacteria and archaea. Our findings suggest that transcription factors can access the chromosome and influence transcription from the membrane in both archaea and bacteria. This study challenges thus the commonly held notion that signal transduction systems require a cytoplasmic transcription factor and highlights the importance of the cytoplasmic membrane in directly influencing signal transduction.

## INTRODUCTION

Signal transduction is the process through which microorganisms regulate their cellular programs according to their extracellular environment. Microorganisms are known to transduce information from outside the cell to the cytoplasm via two-component and one-component signal transduction systems (1, 2). Two-component signal transduction cascades are typically composed of a membrane localized sensor histidine kinase that, when stimulated, transfers a phosphate to a soluble response regulator, resulting in a cellular response (1–3). In contrast, one-component signal transduction systems are composed of a single protein that both directly detects a stimulus and modulates a cellular response (1–3). The vast majority of signal transduction systems in bacteria are one-component systems with most harboring DNA-binding domains and thereby controlling gene expression (2). A majority of one-component regulators are predicted to be localized within the cytoplasm, presumably to have unimpeded access to their DNA target(s) (2). Nonetheless, there are known examples of one-component regulators that are localized to the cytoplasmic membrane (Table 1). Here, we define these one-component regulators and those with similar features (i.e., the presence of a predicted transmembrane [TM] region and a predicted DNA-binding domain) as TM transcription regulators (TTRs). Functional TTRs are found in bacteria and archaea, but not in eukaryotes, due to the separation of the cytoplasmic membrane and their genomes by the nucleus (2, 4, 5). Within archaea, TTRs are known to regulate motility and pilin gene expression in response to dangerous temperatures and nutrient-limiting conditions (6, 7). TTRs are better studied in bacteria, where they have been shown to regulate bile salt resistance (8, 9), toxin production (10, 11), antibiotic resistance (12, 13), acid resistance (14–16), natural competence (17), pilin/fimbria expression (18–24), type 3 secretion systems (25, 26), biofilm formation (27, 28), and metabolism (29–31), and have been implicated in modulation of the human immune system (32) (see Table 1 for additional details).

**TABLE 1:** Characterized TTRs, along with their known cellular responses and associated proteins

TTR	Organism(s)	Cellular response(s)	Associated protein	Reference(s)
ToxR	*Vibrio* spp., *Photobacterium* spp.	Bile salt resistance, cationic antimicrobial peptides, pressure response, biofilm formation, and virulence factor expression	ToxS	8, 9, 11, 19, 27, 77–88
TcpP	Vibrio cholerae and Vibrio fischeri	Virulence factor (*toxT* expression), motility, chemotaxis, and reduction of extracellular polysaccharides	TcpH	18, 89–92
CadC	*Vibrio* spp., Escherichia spp., Salmonella spp., *Yersinia* spp.	Acid resistance	LysP	14, 93–102
TfoS	*Vibrio* spp.	Natural competence	Na	17, 103
VtrA/VttrA	*Vibrio spp.*	Type 3 secretion systems	VtrC	25, 26, 72, 104, 105
VtrB/VttrB	*Vibrio* spp., Salmonella spp.	Type 3 secretion systems	Na	25, 26, 106
MarT	Salmonella spp., Yersinia ruckeri	Fibronectin binding	–^*a*^	107 – 110
GvrA	Escherichia coli	Promotes expression of LEE in response to bicarbonate	Na	111, 112
YqeI	Escherichia coli	Serum resistance, flagellum synthesis, and host cell adhesion	YqeJ	113
PsaE	Yersinia pestis	Fimbria expression	PsaF	20 – 22
MyfE	Yersinia enterocolitica	Fimbria expression	MyfF	23, 114, 115
PypB	Yersinia enterocolitica and Yersinia ruckeri	Flp type IVb pilin expression	–^*a*^	116
BcrR	*Enterococcus* spp., *Lactobacillus* spp.	Bacitracin resistance	Na	12, 13, 117, 118
BreG	*Lactobacillus* spp., *Enterococcus* spp.	Bacteriocin synthesis	Na	119, 120
AguR	*Enterococcus* spp.	Acid tolerance	Na	121 – 124
LP_2991	*Enterococcus* spp., *Lactobacillus* spp.	Immune modulation	Na	32, 125
HcrR	Lactobacillus plantarum	Hydroxycinnamic acid metabolism	Na	29, 126
MmsR	Lactobacillus bifermentans	Isobutyryl-CoA metabolism	Na	30
MtbS	Staphylococcus spp., *Enterococcus* spp., *Lactobacillus* spp.	Virulence factors, phosphate transport, tRNAs, etc.	Na	127
NanR	Staphylococcus spp.	Sialic acid metabolism	Na	31
WmpR	Pseudomonas tunicata	Type IV pilin, pigmentation, iron uptake, amino acid metabolism, biofilm formation, and antifouling	Na	28, 128
ArnR	Sulfolobus acidocaldarius	Motility and pilin expression	Na	6, 7
Rsp	Neisseria gonorrhoeae	Pilin expression	Na	34

a –, There are possible TcpH/ToxS-like genes that are uncharacterized immediately upstream or downstream of the indicated TTR.

Localization to the cytoplasmic membrane has been shown to be critical for some TTRs to influence expression of their target genes (33). TTRs are counterintuitive as their subcellular localization reduces their diffusion and thereby their ability to bind target promoter(s). However, there is a possibility that TTRs evolved from two-component systems. It is known that in Pseudomonas aeruginosa, a two-component system consisting of PilS, the membrane localized histidine kinase, and PilR, the response regulator, regulates activity of RpoN (24). The Neisseria gonorrhoeae genome encodes a chimeric protein called Rsp, which includes the membrane localized receptor of PilS at its N terminus and the PilR DNA-binding domain at its C termini and represses *pilA* expression (34). Bacteroides thetaiotaomicron, a constituent of the human microbiota, contains 32 hybrid histidine kinases, each with a DNA-binding domain within its genome (35). To gain a deeper understanding of TTRs, we performed a large-scale genomic analysis of TTRs across archaeal and bacterial species.

## RESULTS

### Transmembrane transcription regulators are prevalent in bacteria and archaea and display lineage-specific expansion.

**FIG 1:**
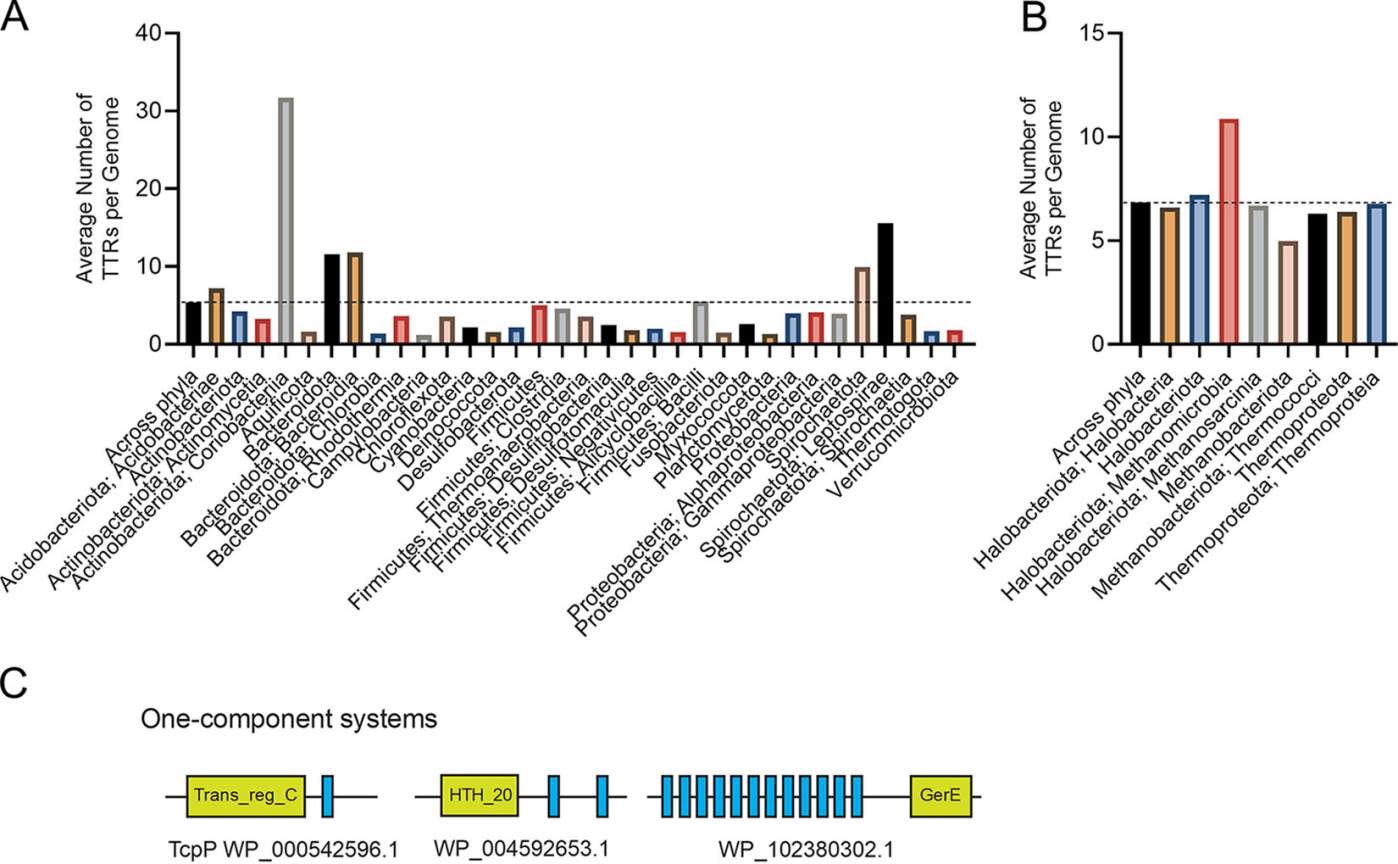
(A and B) Abundance of TTRs among bacterial (A) and archaeal (B) genomes. The dashed line indicates the average number of TTRs per genome across all phyla. Bacterial and archaeal phylum are listed below their respective groups, with the class following a semicolon. To view the supporting data, see File S7 in the supplemental material. (C) Typical membrane topology and domain composition of one-component TTRs identified in this study.

**FIG 2:**
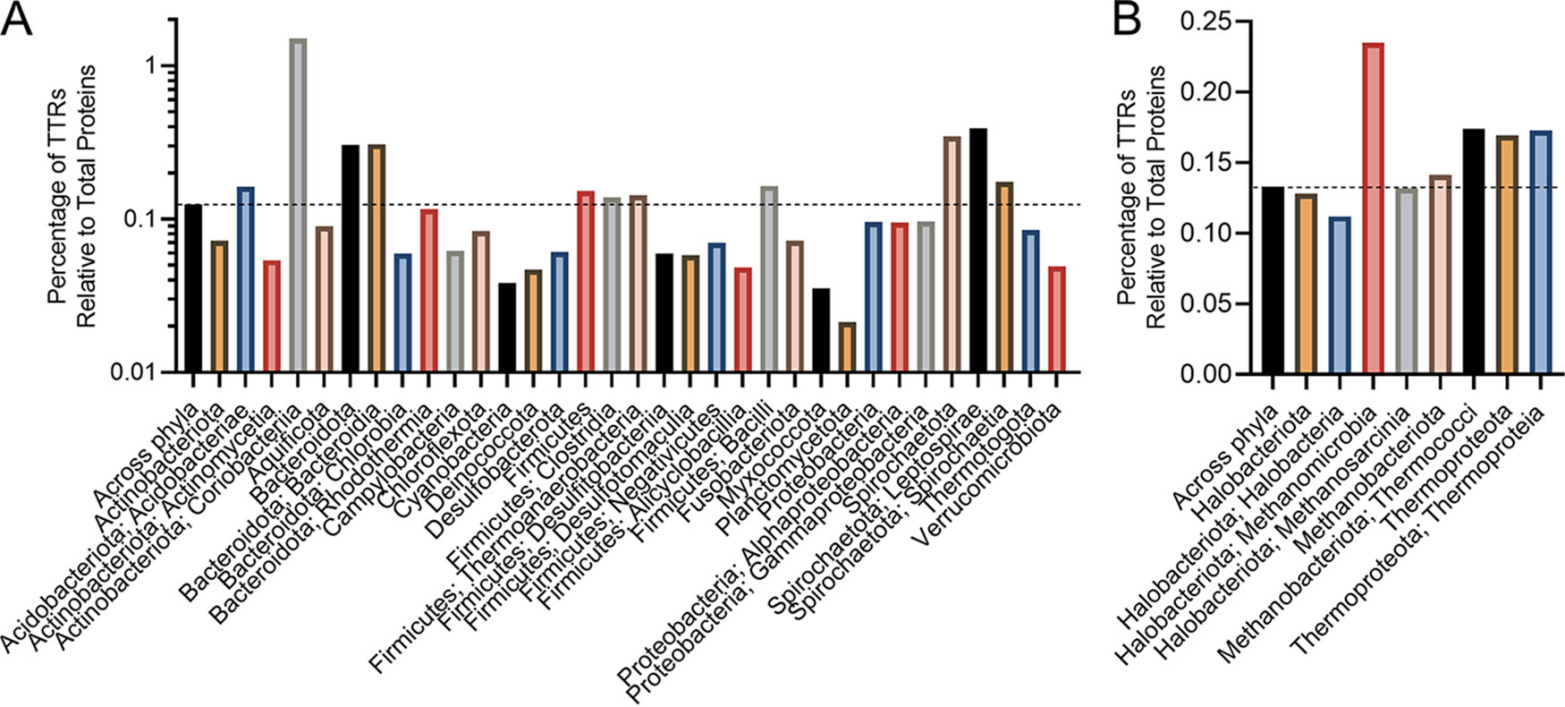
(A and B) Ratio of TTRs to all protein-coding genes among bacterial (A) and archaeal (B) genomes. The dashed line indicates the average ratio of TTRs to protein-coding genes across all phyla. Bacterial and archaeal phyla are listed below their respective groups, with the class following a semicolon. To view the supporting data, see File S7 in the supplemental material.

**FIG 3:**
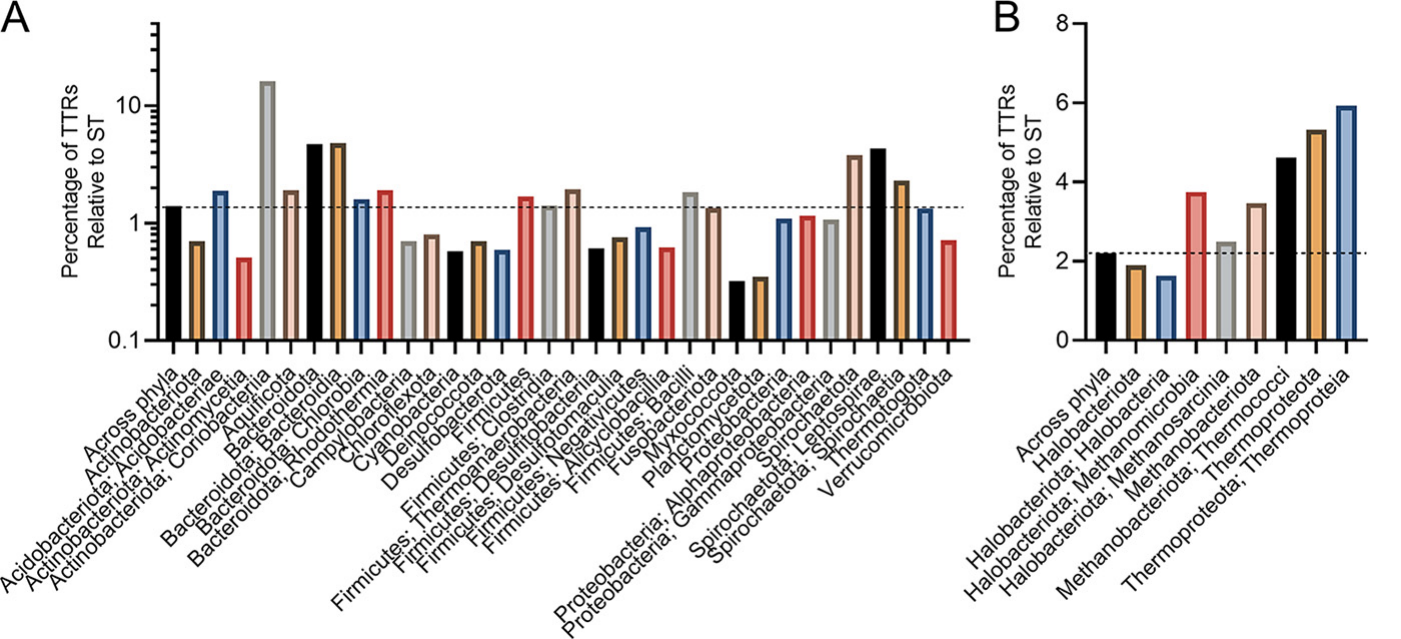
(A and B) Abundance of TTRs relative to signal transduction genes among bacterial (A) and archaeal (B) genomes. The dashed line indicates the average ratio of TTRs to signal transduction genes across all phyla. Bacterial and archaeal phyla are listed below their respective groups, with class following a semicolon. To view the supporting data, see File S7 in the supplemental material.

**FIG 4:**
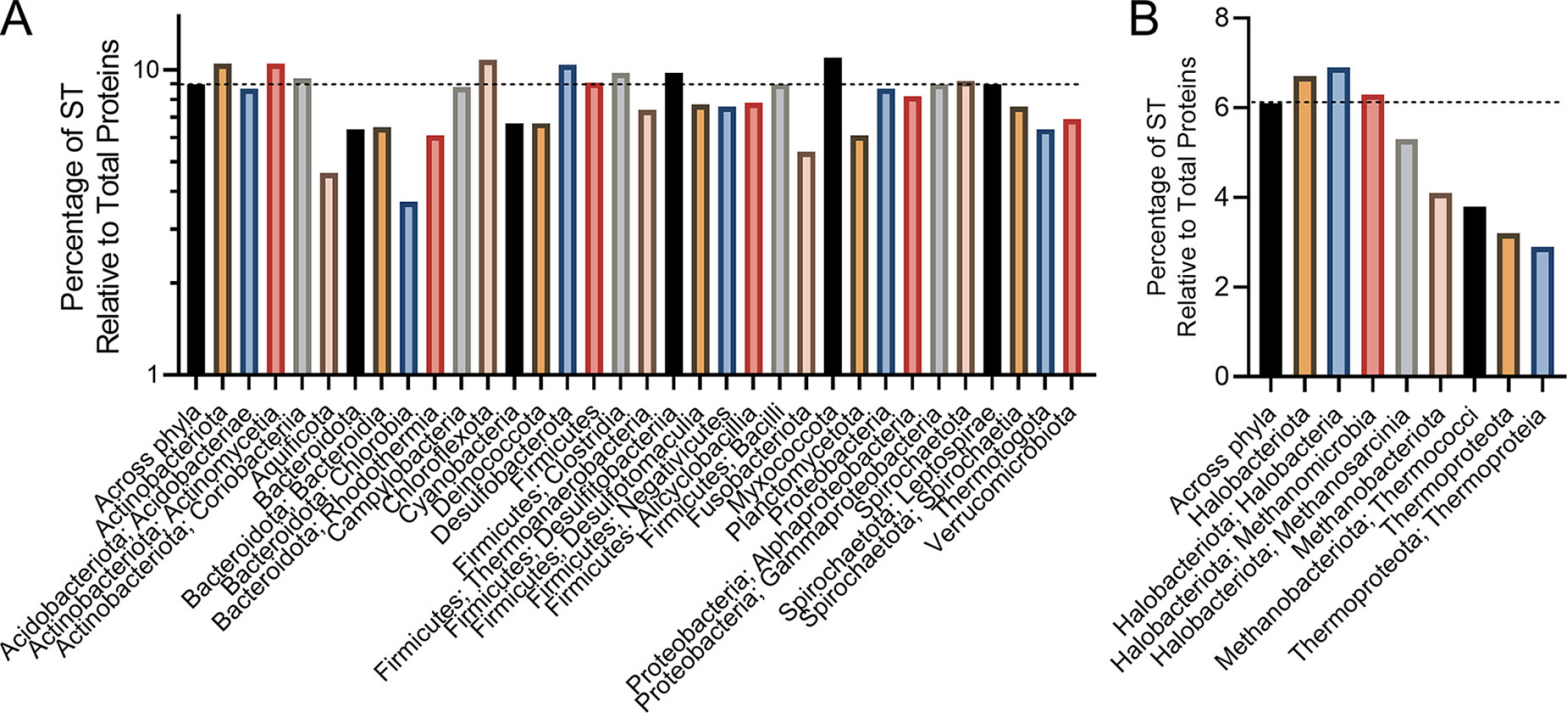
(A and B) Ratio of non-TTR signal transduction genes to total protein-coding genes among bacterial (A) and archaeal (B) genomes. The dashed line indicates the average ratio of signal transduction genes to protein-coding genes across all phyla. Bacterial and archaeal phylum are listed below their respective groups, with the class following a semicolon. To view the supporting data, see File S7 in the supplemental material.

To gain a deeper understanding of the prevalence and distribution of TTRs in prokaryotes, we mined the genomes of 10,933 bacterial and 404 archaeal species for genes that encoded (i) a DNA-binding domain and (ii) at least one transmembrane region. We found that 9,306 bacterial and 367 archaeal species encoded at least one TTR (see File S1 in the supplemental material). In total, we identified 50,302 TTRs (48,918 bacterial and 1,384 archaeal) across 9,673 genomes (see File S1). On average, bacterial genomes contain 5 TTRs (±7) and archaeal genomes contain 4 TTRs (±2) containing one or more TM regions (Fig. 1; see also File S1). Species within the *Coriobacteriia*, *Bacteroidota*, *Bacteroidia*, *Spirochaetota*, *Leptospirae*, *Acidobacteriae*, and *Methanomicrobia* (here and throughout the manuscript: bacterial and archaeal taxonomy is according to Genome Taxonomy Database [36]), on average, contain more TTRs per genome compared to other prokaryotes (Fig. 1A and B; see also File S1). However, the number of TTRs per genome varies dramatically among phyla, with some species encoding only 1 TTR and others encoding up to 158 TTRs (e.g., Raoultibacter timonensis) (see File S1). Given that the genome size also varies dramatically between species (see File S1), we next explored whether the number of TTRs per genome was due to differences in genome size. To address this question, we normalized the number of TTRs to the total number of protein-coding sequences per genome. Across all species, we found that on average 0.15% and 0.13% of bacterial and archaeal coding sequences were composed of TTRs (Fig. 2). This normalization still indicated that species within the *Coriobacteriia*, *Bacteroidota*, *Bacteroidia*, *Spirochaetota*, *Leptospirae*, and *Methanomicrobia* were enriched with TTRs in their genomes. In addition, the *Acidobacteriae*, *Clostridia*, *Thermoanaerobacteria*, *Bacilli*, *Methanobacteriota*, and *Thermoproteota* have a slightly higher abundances of TTRs compared to other bacteria and archaea (Fig. 2). Since TTRs are a distinct type of signal transduction system, we next analyzed what portion of known signal transduction systems within both the bacterial and the archaeal domains were composed of TTRs. Across all species, we found that, on average, 1.5 to 2% of all signal transduction genes were TTRs across bacterial and archaeal species, respectively (Fig. 3). However, these data demonstrate that a high percentage of signal transduction genes are comprised of TTRs within the *Coriobacteriia*, *Bacteroidota*, *Bacteroidia*, *Spirochaetota*, *Leptospirae*, *Methanomicrobia*, *Methanobacteriota*, *Thermococci*, *Thermoproteota*, and *Thermoproteia* (Fig. 3). In addition, the *Acidobacteriae*, *Aquificota*, *Chlorobia*, *Rhodothermia*, *Firmicutes*, *Thermoanaerobacteria*, *Bacilli*, and *Methanosarcina* have a slightly higher percentages of TTRs relative to signal transduction genes (Fig. 3). Given these data, we considered the possibility that the overall abundance of signal transduction genes within bacterial and archaeal phyla and classes could skew our results. To test this, we compared the total numbers of signal transduction genes to the total numbers of protein-coding genes within each bacterial and archaeal species; these values were, on average, 9% for bacterial species and 6% for archaeal species (Fig. 4). Interestingly, we did not observe an increased ratio of signal transduction genes to total protein-coding sequences within the *Coriobacteriia*, *Bacteroidota*, *Bacteroidia*, *Spirochaetota*, *Leptospirae*, *Methanomicrobia*, *Methanobacteriota*, *Thermococci*, *Thermoproteota*, and *Thermoproteia* (Fig. 4). Taken together, these data indicate that the aforementioned bacterial and archaeal clades are indeed enriched with TTRs.

### Transmembrane transcription regulators employ a few common DNA-binding domains.

**FIG 5:**
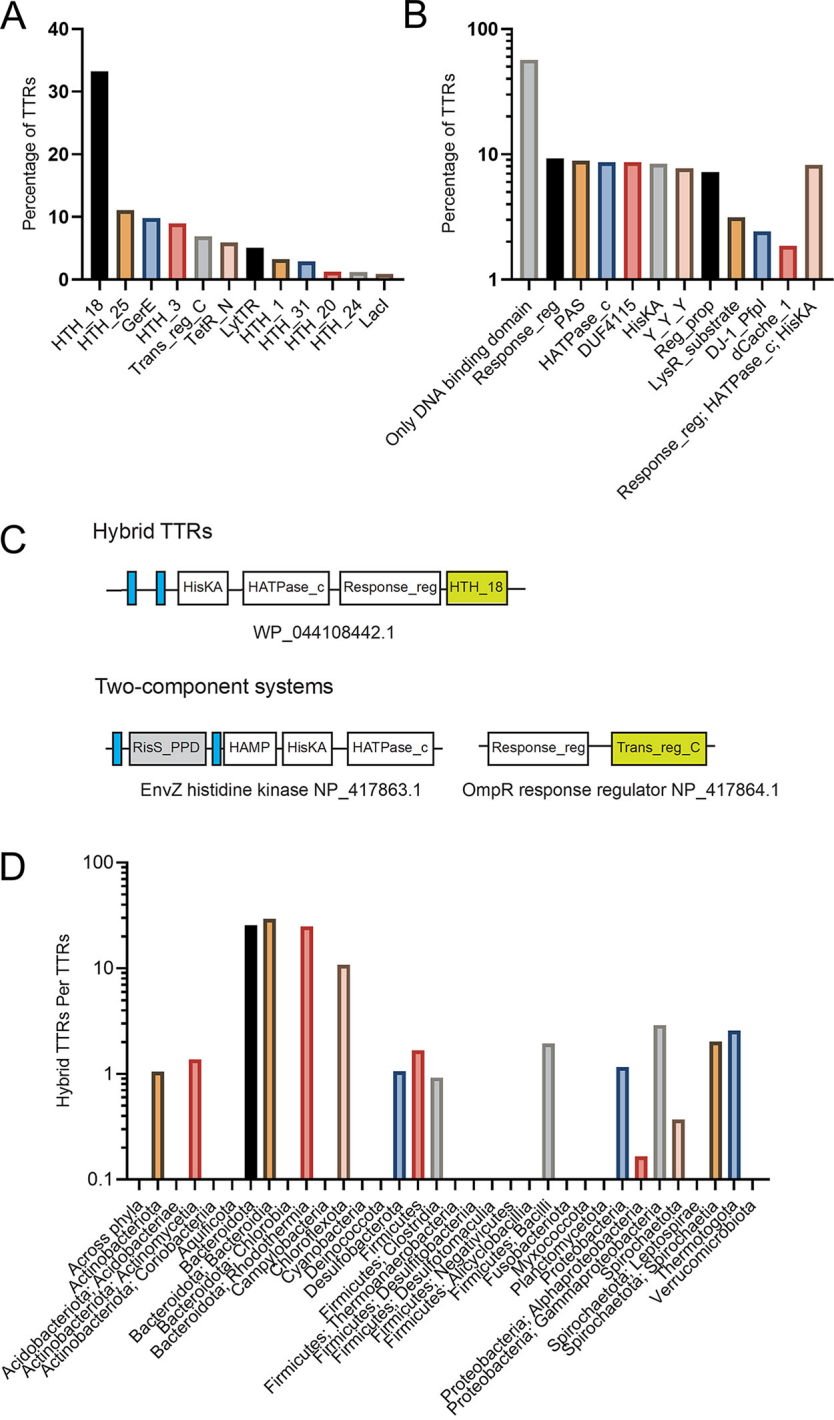
Domain composition of TTRs. (A) Most common DNA-binding domains among bacterial and archaeal TTRs. (B) Most abundant non-DNA-binding domains among bacterial and archaeal TTRs. (C) Typical membrane topology and domain composition of hybrid TTRs and two-component systems. (D) Distribution and ratio of hybrid TTR to TTR across bacterial phyla.

Over a hundred different DNA-binding domains were found across the identified TTRs and only a small number (19 DNA-binding domains) were not found among TTRs (see Table S1A and B in the supplemental material). However, DNA-binding domains of approximately 91% of all TTRs are represented by 11 most common domains, with the helix-turn-helix DNA-binding domain being the most predominant across all TTRs (Fig. 5A; see also Table S1A). A similar distribution of DNA-binding domains has also been reported for other one-component systems (2).

### Hybrid TTRs are unique to bacteria.

Of the domains identified within TTRs, the most common non-DNA-binding domain is the response regulator domain (found in 9.2% of TTRs), which is a part of two-component signal transduction systems (Fig. 5B; see also Table S2) (37, 38). Response regulators catalyze the transfer of a phosphate from a histidine kinase donor and have intrinsic dephosphorylation activity (39). Response regulators are multidomain proteins typically containing a N-terminal receiver domain and a C-terminal effector domain that is commonly a DNA-binding domain (39). Phosphorylation of the receiver domain stabilizes a conformation that allows for activity of the effector domain (39). In addition, among the top five most common non-DNA-binding domains in TTRs are the HATPase_c (a histidine kinase catalytic domain [found in 8.6% of TTRs]), HisKA (a histidine kinase dimerization domain [found in 8.4% of TTRs]), and the Y_Y_Y domains (an extracellular domain found in two-component systems [found in 7.7% of TTRs]) (Fig. 5B; see also Table S2). These domains are typical for two-component signal transduction pathways (37, 38, 40, 41). In fact, approximately 8.2% of all TTRs identified by our analysis contain one or more core domain of two-component systems (response regulator, HATPase_c, and HisKA domains), which we refer to as hybrid TTRs (Fig. 5B and C; see also Table S2). Hybrid TTRs appear to be a bacterial phenomenon, since we found no archaeal TTRs containing a response_reg domain and very few containing a HisKA domain (17 TTRs) or HATPase_c domain (1 TTR).

Bacteroides thetaiotaomicron was reported to contain 32 hybrid histidine kinases with DNA-binding domains (i.e., hybrid TTRs) (35). Our data indicate that a majority of the TTRs within the *Bacteroides* genus are hybrid TTRs (approximately 72%) (see File S3). Furthermore, in addition to the *Bacteroides* genus, many other species within *Bacteroidota*, *Bacteroidia*, *Rhodothermia*, and *Chloroflexota* contain a high fraction of hybrid TTRs (Fig. 5D). Our data indicate that hybrid TTRs may have evolved from canonical two-component regulatory systems, similar to hybrid two-component regulatory systems, that evolved from canonical two-component systems (42). Our data suggest that hybrid TTRs are the product of recent evolutionary events since they are not conserved at the genus, class, or phylum level.

### Single pass transmembrane regulators are the most prevalent TTRs.

**FIG 6:**
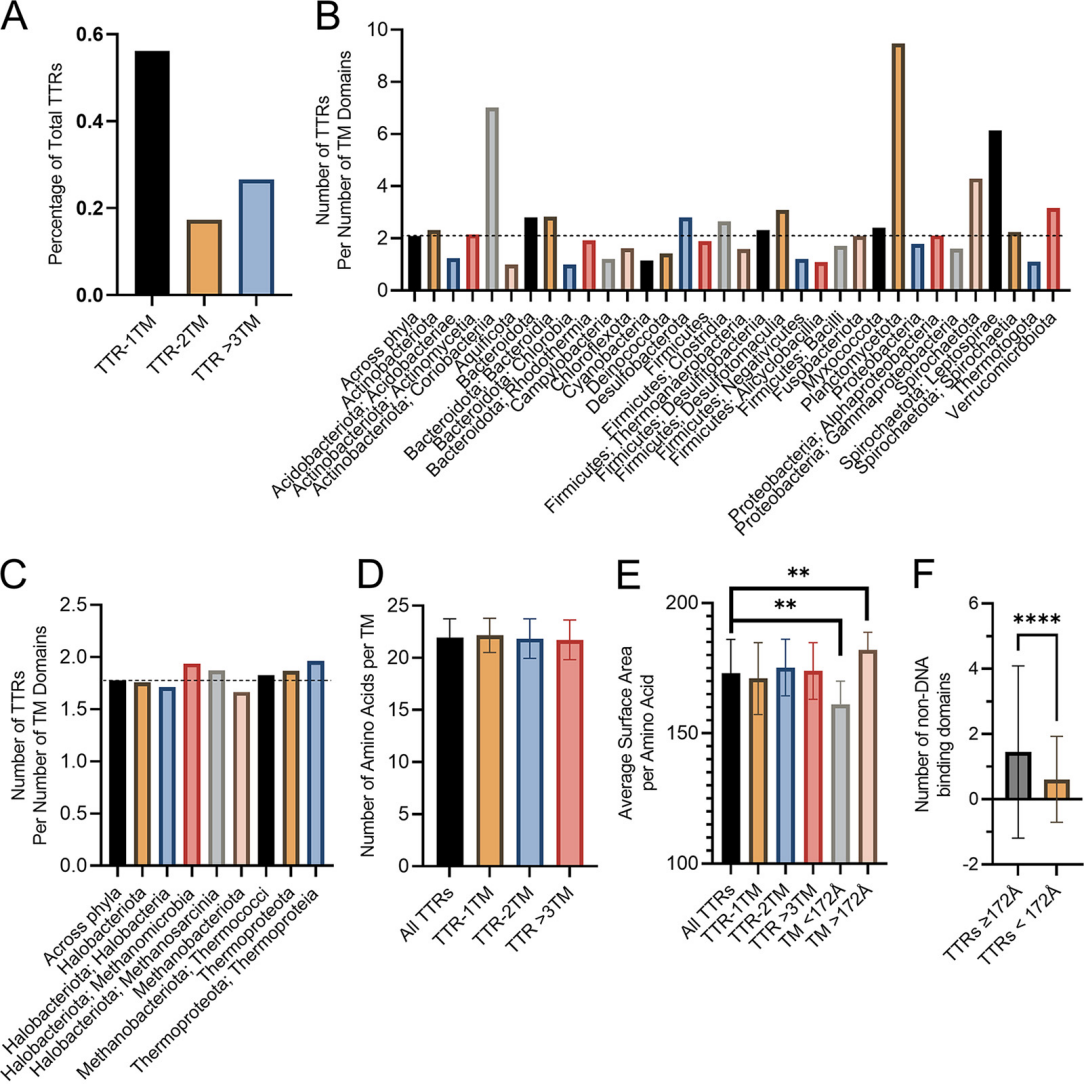
TM region analysis of TTRs. (A) Number of TM regions across all bacterial and archaeal TTRs. (B) Average number of TM regions per TTR within the bacterial phyla. (C) Average number of TM regions per TTR within the archaeal phyla. (D) Average TM region length for TTRs across all bacterial and archaeal species. (E) Average surface area of TTR TM region among bacterial and archaeal species. (F) Average number of non-DNA-binding domains per TTR based on the average surface area of the TM region. Error bars represent the standard deviations. **, *P* < 0.01; ****, *P* < 0.0001. A two-tailed unpaired Student *t* test was used to determine statistical significance.

We found that approximately 56% of TTRs are single-pass transmembrane (TM) proteins, while TTRs containing two or more TM regions comprise 17 and 27% of TTRs, respectively (Fig. 6A; see also Files S4 to S6). We next questioned whether TM content of TTRs was homogenous across bacterial and archaeal phyla. Since some TTRs contain up to 22 TM regions, the average number of TM regions per TTRs, approximately 2 TM regions, is elevated within bacterial and archaeal phyla despite that the majority of TTRs are single-pass TM proteins (Fig. 6B and C; see also Files S4 to S6). The average number of TM regions per TTRs is consistent across archaeal phyla and classes while there is greater diversity among bacterial phyla. In bacteria, *Coriobacteriia*, *Planctomycetota*, *Leptospirae*, and *Verrucomicrobiota* contain TTRs with three or more TM domains on average (Fig. 6B and C).

### TTRs have a propensity to associate with liquid-ordered membrane domains.

We hypothesized that TTRs may respond to their local membrane environment which can be influenced by extracellular conditions. A common phenomenon across bacteria and eukaryotes is the formation of small (i.e., nanometer) lipid regions, referred to here as lipid rafts (43–45). Lipid rafts and non-lipid-raft membrane regions have been shown to influence various cellular processes, including signal transduction, in part due to their ability to promote interaction between membrane localized proteins (46–55). Association with lipid raft and non-lipid-raft membrane regions is determined by the properties of the transmembrane region with length and overall surface area of the transmembrane helix being the most critical factors (56). Given that lipid rafts were found in bacteria, we asked a question whether TTR TM regions supported their association with lipid raft or non-lipid-raft membrane regions. Since the overall length and surface area of transmembrane helices controls the association of membrane proteins within lipid raft membrane regions, we calculated the overall length and surface area of all predicted transmembrane regions for TTRs analyzed here (see Files S4 to S6). Overall, we found that the TM region length and surface area remain similar across single TM or multi-TM region TTRs (Fig. 6D and E; see also Files S4 to S6). We found that a substantial number of TTR TM regions (approximately 43%) have a surface area equal to or below 172 Å per amino acid (Fig. 6E; see also Files S4 to S6). Prior studies have demonstrated that TcpP, a TTR that positively modulates virulence in Vibrio cholerae, increases its association with detergent-resistant membranes (i.e., liquid-ordered membrane domains) in the presence of ɑ-linolenic acid, a dietary fatty acid (57). The surface area of the TcpP transmembrane region is 172 Å per amino acid. These data suggest that many TTRs might have the capacity to associate with liquid-ordered membrane domains. Furthermore, this suggests that TTRs might be directly modulated by the cytoplasmic membrane. We reasoned that if the cytoplasmic membrane is a major signal for TTRs with low TM surface area that they would also contain fewer sensory domains (i.e., non-DNA-binding domains). Indeed, we found that TTRs with a surface area equal to or below 172 Å per amino acid contained fewer sensory domains compared to TTRs with a higher TM surface area (Fig. 6F).

## DISCUSSION

Our analysis revealed that TTRs are widespread in bacteria and archaea and in some genomes, for example *Raoultibacter timonensis*, they are a major type of transcription regulators (41% of all DNA-binding transcription factors). We found that TTRs are abundant in species from the gut microbiota. For example, species within the *Eggerthella* genus (the *Coriobacteriales* order), contain a high number of TTRs per genome (~117 TTRs) (see File S1 in the supplemental material). The *Eggerthellaceae* family are common members of mammalian gastrointestinal tracts (58–62). Members of the *Bacteroidia*, commonly associated within the human gastrointestinal tract, were also found to contain a high number of TTRs and hybrid TTRs within their genomes (Fig. 1–4). There is evidence that hybrid TTRs within *Bacteroides* genus function to sense and respond to disaccharides (63). It remains to be seen whether TTRs within the *Coriobacteriales* and *Bacteroidota* contribute to their ability to colonize mammalian gastrointestinal environments, but our data have revealed this correlation.

The advantage of TTRs versus classical one- and two-component systems is not obvious: why bring the response regulator and DNA-binding domain to the cytoplasmic membrane? One possibility is that the membrane itself serves as a signal to further fine tune these signal transduction pathways. It is generally recognized that the membrane environment is not a homogenous. For example, a vast majority of integral membrane proteins are heterogeneously distributed in Bacillus subtilis cells, indicating that their diffusion within the cytoplasmic membrane is limited (64, 65).

Both bacterial and eukaryotic cells support the formation of small-lipid-raft membrane regions (43–45). Generally speaking, lipid raft and non-lipid-raft membrane regions differ by their overall fluidity and thickness, with lipid rafts having a lower fluidity and increased thickness, as a consequence of the phospholipid species that occupy these membrane environments (45, 46, 66–69). These membrane regions have been shown to influence many signaling pathways in eukaryotic cells, in particular T cells (46–55). There is also evidence that dietary polyunsaturated fatty acids can influence formation and stability of lipid ordered membrane domains thereby influencing signal transduction (48, 49). Here, we demonstrate that a large fraction of TTRs (approximately 43%) can associate with lipid raft membrane regions. These data support our hypothesis that the membrane environment may serve to influence TTR function; however, additional studies are required to further test this hypothesis.

In addition to the potential influence of the cytoplasmic membrane, it is also feasible that localization of TTRs to the cytoplasmic membrane facilitates tighter regulatory controls on TTR regulons. That is, not only do TTRs require a signal to impact gene expression, but that the target promoter sequence(s) must be in close proximity to the cytoplasmic membrane. This would imply that the structure of the chromosome (which is influenced by nucleoid associated proteins, transcription, translation, DNA supercoiling, and condensins) also contributes to TTR gene regulation. This would indicate that TTRs could contribute to regulation of complex lifestyles among microorganisms. Within the *Alphaproteobacteria*, a family of LytTR response regulators, not exclusively TTRs, were identified (70). Curiously, within *Caulobacter cresentus* all instances of the LytTR response regulator domain contain one or more TM regions (70). This could explain the high degree of variation of TTRs across the bacterial and archaeal domains.

Some TTRs (such as ToxR, VtrA, VtrrA, PsaE, MyfE, TcpP, and YqeI) have an associated protein that contributes to TTR mediated signal transduction by inhibiting proteolysis of the TTR or by stimulating heterodimer formation (Table 1). These TTRs are also referred to as cocomponent systems (71). Curiously, many of these associated proteins contain a lipocalin-like domain, which has been shown to be important for VtrC binding to bile, but generally are known to bind to small hydrophobic molecules (71, 72). Rather than responding to the cytoplasmic membrane itself, localization of TTRs to the cytoplasmic membrane may facilitate function of cocomponent systems.

Our data suggest that TTRs play a much larger role in signal transduction than previously thought, implying that transcription initiation also occurs at the membrane-DNA interface across bacterial and archaeal species. However, compared to the number of TTRs identified by our analysis the number of experimentally validated TTRs is extremely low indicating that a large fraction of TTRs function remains to be understood (Table 1). In support of this, a substantial fraction of TTRs identified here (~57%) contains only a DNA-binding domain and no additional recognizable domains of known function (Fig. 5B). Thus, this work provides new insights into the prevalence and distribution of TTRs within bacteria and archaea reveals unique features of these regulators.

## MATERIALS AND METHODS

### Identification and transmembrane domain analysis of TTRs within the MiST database.

TTRs for a representative set of genomes were collected from the MiST database by running a custom Python script on the local computer cluster (73). The script, which is available in the GitHub repository (https://github.com/bioliners/TTRs), sends requests through the database API and processes the results. For each genome, all DNA-binding signal transduction proteins that contain transmembrane regions were retrieved (see File S2 in the supplemental material). The Pfam Profile hidden Markov models of DNA-binding domains listed on the help page of the MiST database were used to identify DNA-binding domains in the proteins. All DNA-binding domains identified in TTRs and those that were checked but not identified are listed in Table S1. TM regions of the protein sequences were identified by running TMHMM, domains were verified using TREND and Pfam profile hidden Markov models (74–76). Sequences corresponding to transmembrane regions were extracted using a custom Python script. The average length, number of amino acids, and surface area for each TTR transmembrane region were calculated from data in Files S4 to S6 using a Python script. Taxonomy information for the genomes was retrieved from GTDB database (36; see File S1 for data). All used scripts are available in a GitHub repository (https://github.com/bioliners/TTRs).

### Calculating statistical parameters of TTRs.

The total number of all signal transduction (ST) proteins encoded in each analyzed genome was obtained from MiST database (73). The genome size and the number of all encoded proteins were obtained from the metadata tables of GTDB (36; see Files S1 and S7 in the supplemental material). These data were cross-referenced with the number of encoded TTRs in each genome using Excel. For statistical analysis, we considered phyla or classes that contained at least 10 sequenced genomes. By running Shapiro-Wilk test and exploring quantile-quantile plots, we have established that TTR counts are not normally distributed. Therefore, we used two nonparametric tests, Spearman and Kendall correlation tests, to explore correlations. Prior to performing correlation tests, we subtracted TTR counts from the overall counts of ST proteins and ST protein counts from the sum of all proteins encoded in each genome. This was done to remove the contribution of correlation of TTR and ST proteins with themselves. Statistical analysis was carried out in the R environment.

### Data availability.

The data presented here are available from the corresponding authors upon request.
